# Integration of Health Education and Promotion Models for Designing Health Education Course for Promotion of Student’s Capabilities in Related to Health Education

**Published:** 2018-09

**Authors:** Davoud SHOJAEEZADEH, Hashem HESHMATI

**Affiliations:** 1. Dept. of Health Education and Promotion, School of Public Health, Tehran University of Medical Sciences, Tehran, Iran; 2. Dept. of Public Health, School of Health, Torbat Heydariyeh University of Medical Sciences, Torbat Heydariyeh, Iran

## Dear Editor-in-Chief

Despite importance of health education, there is a lack of health education by scientific method in health system because of lack of adequate capabilities and competencies in health system staffs. On the other hand, traditional educational methods have not adequate effectiveness for creating capabilities and behavior change (1–3). We aimed to assess the integration of health education and promotion models for designing health education course for promotion of student’s capabilities in related to health education.

This qualitative study was done during 2013 to 2016 among 132 public health students in Iran that were selected by using census method. This study was conducted in three dimensions: Curriculum design and revision, teaching methods and application of techniques and assessment, evaluation and educational effectiveness. For designing health education course were used an integration of health education and promotion models such as health belief model, PERECEDE, and BASNEF models. Focus group discussion was used for evaluating the program. Assessment of the study was done through observation and checklist. Data were analyzed through content analysis.

The results have been set in the three-part including student, health systems, research and scientific publishing. The most important result of the study was as follows: Knowledge, attitude and skills of students in health education were increased. Promoting participation and involvement of the students in educational classes by active learning, Promoting empowering of students in team work, Promoting interaction between professor and students, Improvement and modification of students attitudes in related to educational major, health education course and educational climate and Improvement self-efficacy in the students. Empowerments of health system staffs were increased. Number of student’s research was increased (4, 5).

Integrated model led to promotion of student’s capabilities but using quantitative methods along with qualitative methods can evaluate the efficacy of the model more specifically. According to the efficacy of the integrated model, we recommended the model were used for teaching health education course and other similar courses.
